# Differential Expression of microRNAs Correlates With the Severity of Experimental Autoimmune Cystitis

**DOI:** 10.3389/fimmu.2021.716564

**Published:** 2021-07-14

**Authors:** Vijay Kumar, Sonia Kiran, Haidar A. Shamran, Udai P. Singh

**Affiliations:** ^1^ Department of Pharmaceutical Sciences, College of Pharmacy, The University of Tennessee Health Science Center, Memphis, TN, United States; ^2^ Pathology, Microbiology, and Immunology, School of Medicine, University of South Carolina, Columbia, SC, United States

**Keywords:** microRNAs, autoimmune cystitis, inflammation, urinary bladder, cytokine

## Abstract

Interstitial cystitis (IC)/bladder pain syndrome (BPS) primarily affects women. It varies in its severity and currently has no effective treatment. The symptoms of IC include pelvic pain, urgency and frequency of urination, and discomfort or pain in the bladder and lower abdomen. The bladders of IC patients exhibit infiltration by immune cells, which lends credence to the hypothesis that immune mechanisms also play a role in the etiology and pathophysiology of IC. The Differentially expressed microRNAs (miRs) in immune cells may serve as crucial immunoregulators in the IC. Therefore, we sought to determine whether miRs might play a regulatory role in the progression and pathogenesis of IC, using experimental autoimmune cystitis (EAC) model. In the present study, we observed differential expression of a specific subset of miRs in iliac lymph nodes (ILNs) and urinary bladders (UB) of IC mice compared to that in control mice. Microarray analysis of 96 miRs from the bladder and 135 miRs from ILNs allowed us to identify 50 that exhibited at least a 1.5-fold greater difference in expression in EAC mice compared to control mice. Hierarchical cluster analysis of the microarray data was used to search available databases to predict molecular pathways with which the miRs might interact. Four miRs from each organ that exhibited altered expression in EAC mice and that were predicted to have roles in inflammation (miR-146a, -181, -1931, and -5112) were selected for further analysis by reverse transcription-polymerase chain reaction (RT-PCR). All were confirmed to be elevated in EAC mice. Histological inflammatory scores, systemic chemokines, and cytokines expressed by T helper type 1 (Th1) lymphocytes were also elevated in EAC mice as compared to control animals. We hypothesize that the mechanism of EAC induction might involve the modulation of specific miRs that increase local and systemic levels of chemokines and cytokines. The present study identifies novel miRs expressed in UB and ILNs that will allow us to highlight mechanisms of EAC pathogenesis and may provide potential biomarkers and/or serve as the basis of new therapies for the treatment of IC.

## Introduction

Interstitial cystitis (IC), also known as bladder pain syndrome (BPS), affects 3.3 to 7.9 million women in the US, a rate 5 times greater than that observed for men, yet its cause remains unknown (1). Many IC patients suffer from psychological stress, anxiety, depression, insomnia, pain, and negative performance. Despite a decade-long effort by the National Institutes of Health (NIH) and other organizations, little progress has been made on developing effective treatments for IC. The development of future treatments will rely on better clarification of the mechanisms and pathogenesis of IC. Current animal models have advanced our understanding of IC pathogenesis, but no model of bladder injury in healthy animals completely mimics the salient features of human IC. Therefore, the development of improved animal models for IC is a specific need that will greatly advance our understanding of underlying IC disease mechanisms and aid in identifying potential novel preventative measures, better diagnostic tools, and safe and effective therapeutic options. The discovery of novel methods of prevention and treatment for IC that are safe and effective would have a tremendous economic effect of >$66 billion (USD) in the U.S. alone (2).

The lack of experimental models that manifest clinical features of human IC hampered research in this area until recently. Given the apparent link between IC and inflammation, we generated experimental autoimmunity models in mice that feature pro-inflammatory T helper 1 cells (Th1) targeted against a specific self-antigen. This concept underlies the creation of useful models for autoimmune encephalomyelitis (EAE) (3), myocarditis (4), and oophoritis (5). To create an autoimmune model for IC, we immunized male and female SWXJ mice with a lyophilized bladder homogenate from SWXJ mice to induce nonspecific systemic bladder autoimmunity and autoimmune complications. The resulting murine experimental autoimmune cystitis (EAC) model is a significant advance in this research field that displays an IC-like phenotype of urinary frequency, pelvic nociception, and histological alterations in the urinary bladder. These alterations represent many of the clinical characteristics of human IC (6), including urinary frequency, decreased urine output per void, several histopathological features such as urothelial cell detachment, and increased bladder permeability with epithelial leakage (7).

The principal etiology of IC appears to involve an increase in the urinary bladder’s local immune response, urinary antibody concentration, and numbers of immune cells (8). In particular, there is an increase in the numbers of mast cells that accumulate below the epithelial layer during experimental and clinical IC (9), producing a variety of cytokines and chemokines that attract neutrophils (10) and T cells (11). Determining the precise role of autoimmunity during IC progression will result in the development of better treatment options. To this end, the detection of differential expression of microRNAs (miRs) in naïve, activated and effector immune cells suggest that miRs may play key roles in immune-cells effector function. It has been shown that miRs are differentially expressed in the human colon during ulcerative colitis (UC), where they serve as negative regulators of inflammation and innate immunity (12). miRs processing is also impaired in conditional KO Dicer mice, which exhibit a defect in T cell development and Th cell differentiation with Th1 polarization (13). Additionally, we have shown that dysregulation of miR-155 induces Th1/Th17 polarization and protects mice from inflammation (14). To the best of our knowledge, no information to date is available for any role of urinary bladder (UB) miRs in an experimental model of IC.

We have shown that CD4^+^ T cells, mast cells, and neutrophils infiltrated in the UB in two different cyclophosphamide (CYP)-induced models for autoimmune IC, and blocking these pathways reduces the symptoms of IC (7, 15). To extend this study to determine a possible mechanism of miRs action in IC development and progression, we set out to identify and verify the altered set of immune cells derived differential miRs expression in ILNs and UB that might modulate the progression of IC. The identification of specific miRs involved in this process would represent a major leap forward for the IC field. Even more exciting, our data suggest that targeting these sets of altered miRs might represent a novel approach to the prevention and treatment of IC. The findings from this study will help to develop improved treatment protocols for IC and justify future correlative studies to validate the levels of specific miRs for use as a non-invasive biomarker for IC-like conditions.

## Materials and Methods

### Animals and Ethics Statement

Female SWXJ mice (6-8 weeks old) were purchased from Jackson Laboratory (Bar Harbor, ME, USA) and brought to the University of South Carolina (USC) School of Medicine animal facility. The Jackson laboratory generates SWXJ (H-2^QS^) mice by mating SJL/J (H-2^s^) males with SWR/J (H-2^q^) females and houses them for six weeks before shipment. At the USC facility animals were housed and maintained in isolator cages under normal light and dark cycles in conventional housing conditions to minimize animal pain and distress. The Institutional Animal Care and Use Committee (ICAUC) of the University of South Carolina School of Medicine approved this study from 2014 to 2017. Dr. Singh and a resident veterinarian monitored the pain and distress of mice each day and were prepared to administer analgesics if they had been necessary. Experimental groups consisted of six mice (n=6/group) and each study was repeated three times. In this study, we used a total of 46 mice including 10 mice for making bladder homogenate.

### Induction of Experimental Autoimmune Cystitis (IC)

The urinary bladder **(**UB) from 8-10-week-old female SWXJ (H-2^q.s^) mice were homogenized in double-distilled deionized water (DW) using a hand tool (Fisher Scientific, USA). The homogenate was centrifuged at 1000g for 10 minutes and the supernatant was lyophilized overnight to reduce the volume of each bladder homogenate to around 100 μL. These homogenates were used to immunize 8-10-week-old female SWXJ mice subcutaneously in the abdominal flank with complete Freund’s adjuvant (CFA) containing 400 μg of *Mycobacterium tuberculosis* H37RA (Difco, Detroit, MI). A group of similar 8-week-old female mice was immunized with an emulsion containing CFA alone to serve as controls. These mice develop EAC four months after the transfer of the UB homogenate (6). Mouse behavior (pain, grooming, guarding) that could reflect symptoms of IC were monitored twice a week until the experimental endpoint (data not shown). After the experimental endpoint, mice were euthanized by an overdose of isoflurane. Blood was collected to determine levels of systemic chemokines and cytokines by ELISA.

### Cell Isolation

At the experimental endpoint, single-cell suspensions were prepared from the iliac lymph nodes (ILNs) and UBs obtained from each group of mice. Cells were dissociated and RBCs lysed with lysis buffer (Sigma St. Louis, MO). After centrifugation, single-cell suspensions of ILNs and UB were passed through a sterile filter (Sigma St. Louis, MO) to remove any debris. Single-cell suspensions were washed twice in RPMI 1640 (Sigma St. Louis, MO) and stored in TRIzol reagent for isolation of total RNAs, as described below.

### miRs Isolation and Microarray Analysis

Cells from ILNs and UB were immediately placed in TRIzol reagent obtained from a miRNeasy mini kit (QIAGEN, Valencia, CA). miRs were isolated according to the manufacturer’s instructions and samples were stored at -80^0^C before being sent to the John Hopkins University sequencing core facility for microarray analysis. Total RNA from ILNs and UB including miRNAs was hybridized to an Affymetrix GeneChip high-throughput miR array containing 609 murine probes (Affymetrix, Santa Clara, CA). The data generated from the array were analyzed using hierarchical clustering. All miRs raw data were submitted to Gene Expression Omnibus (Accession number **GSE177066**; control mice **GSM5373940** and EAC mice **GSM5373941**). By use of ingenuity pathway analysis (IPA) software (Qiagen; www.ingenuity.com), the results from the miRs microarray were analyzed to identify molecular pathways potentially altered by single or multiple miRNAs target genes. In brief, this analysis compares each set of miRs to all available pathways in the database and assigns priority scores based on the predicted strength of the miRs interaction with components of the target pathway.

### Validation of miRs Data With RT-PCR

To validate key results from the microarray analysis, four representative miRs from bladder tissue and ILNs (miRs-146a, miRs-181, miRs1931, and miRs-5112) were significantly altered based on microarray results and relevant to inflammation were subjected to further RT-PCR analysis as described in our previous study (16). In brief, miRs were reverse-transcribed (miScript II; Qiagen) to make the first-strand cDNA that was subsequently analyzed by quantitative RT-PCR with primers specific for miRNAs 455 and 101b, respectively (5′ GCAGUCCACGGGCAUAUACAC-3′ and 5′-UACAGUACUGUGAUAGCUGAA-3′). Reactions were performed with SYBR Green Master Mix (Qiagen). The analysis involved initiation at 95°C for 15 minutes then 40 cycles of 94°C for 5 seconds, 55°C for 30 seconds, and 70°C for 30 seconds. All samples were analyzed in triplicate.

### Cytokine Measurement by Luminex™ Analysis

Levels of T helper cell-derived cytokines IL-6, MCP-1, INF-γ, TNF-α, CXCL9, and CXCL10 in the serum were determined using a Luminex plex Elissa assay kit (Millipore, Sigma USA). We previously described the detailed method elsewhere (14). In brief, assay buffer containing beads specific for these cytokines and 50 µl of assay beads were added into pre-wet vacuum wells, the assay buffer was removed, and the plate was washed with wash buffer. We added 50 µl of the sample (standard, blank, or mouse serum) to each well and incubated for 1 hour with continuous shaking using a Lab-Line™ Titer Plate Shaker (Melrose, IL). The filter bottom plates were washed and vortexed at 300x g for 30 seconds. Subsequently, 25 µl of anti-mouse detection antibodies were added to each well and incubated for 30 minutes at room temperature. Next, 50 µl of the streptavidin-phycoerythrin solution was added to each well and incubated with continuous shaking for 10 minutes at RT. Finally, 125µl of assay buffer was added to each well, and fluorescence was measured using a Luminex™ System (Austin, TX) and calculated using BioRad software. The Ab BioRad assays were capable of simultaneously detecting >10 pg/ml of each analyte (IL-6, MCP-1, INF-γ, TNF-α, CXCL9, and CXCL10).

### Histopathological Examination

Mouse urinary bladders (UBs) were subjected to histopathological examination to detect inflammatory cell infiltrates and signs of IC pathology. UBs were preserved using 10% neutral formalin for 24 hrs and embedded in paraffin. Fixed tissues were sectioned at 6 µm, stained with hematoxylin and eosin, and examined by light microscopy. The inflammatory state of each UB was characterized and scored as follows: having no change when compared with tissue samples from control mice (score = 0); having a few mononuclear cell infiltrates (score = 1); having minimal hyperplasia with a mixture of mononuclear cells (score = 2); having major hyperplasia (score=3) or having major hyperplasia with heavy cellular infiltrates in the sub-mucosa (score = 4).

### Statistical Analysis

The traditional α-value (*P* < 0.01) was used to evaluate statistical significance in this study. The data are expressed as the mean ± SD and were compared using a two-tailed paired student’s *t*-test or an unpaired Mann-Whitney *U*-test. The results were analyzed using Microsoft Excel (Microsoft, Seattle, WA) for Macintosh computers. Single-factor and two-factor ANOVA analyses were used to evaluate groups and subgroups, respectively. The Kolmogorov-Smirnov (K-S) two-sample test using CXP analysis software (Beckman Coulter, USA) was used to compute the statistical significance between histograms.

## Results

### Changes in miRs Expression in Immune Cells During Experimental IC

**Figure 1:**
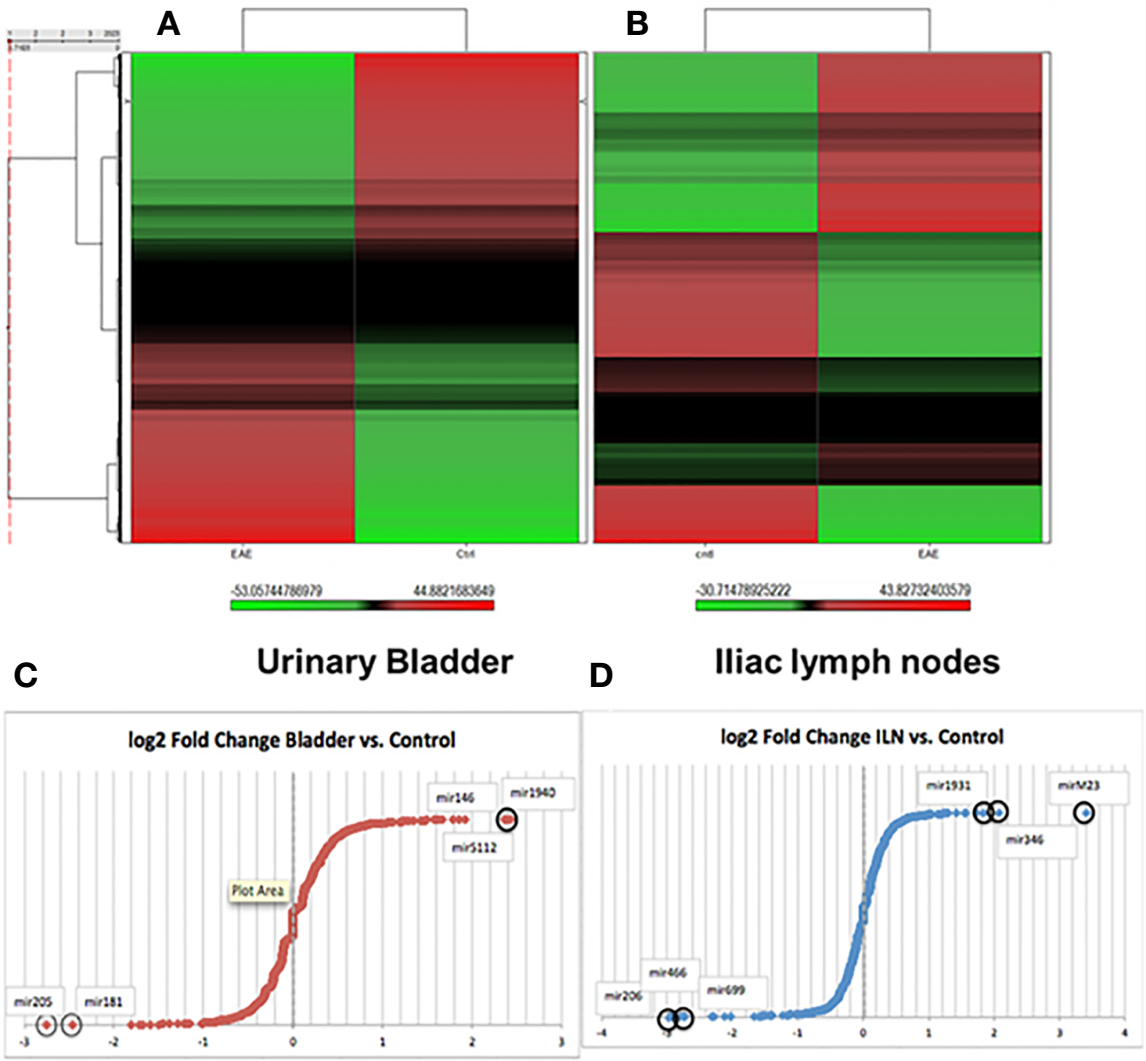
Changes in unsupervised hierarchical clustering of differentially expressed miRs in a mouse model of IC *versus* normal controls between the urinary bladder and iliac lymph nodes tissue derived immune cells reveal divergent pathways. **(A)** Heat map of the miRs array done on immune cells from urinary bladder *versus* control. **(B)** Iliac lymph nodes *vs.* control. Red color indicates an upregulation, green color indicates a downregulation, and black color indicate no change. **(C)** Fold changes in urinary bladder normalized fold change (FC) in a mouse model of IC in Log2 scale. **(D)** Fold changes in iliac lymph nodes miRs expression normalized FC in a mouse model of IC in Log2 scale.

The function of various genes can be altered by epigenetic regulation mediated by altered miRs expression. To further define the mechanisms of gene regulation during IC progression, we isolated total RNAs from immune cells harvested from ILNs and UB and performed miRs array analysis in the core facility at the Johns Hopkins School of Medicine, MD. **Figures 1A**, **B** depicts the heat map illustration of hierarchical clustering analysis based on differentially expressed miRs in the ILNs and UB. This image was generated as a tree showing significant differences between miRs isolated from IC and control mice. We observed a distinct set of altered miRs expression patterns in the mice with IC as compared to the controls, suggesting that dysregulation of miRs in the UB and ILNs may play a role in the development or progression of IC. To the best of our knowledge, this is the first report on changes in miRs expression in UB and ILNs in an experimental mouse model of IC.

**Figure 2:**
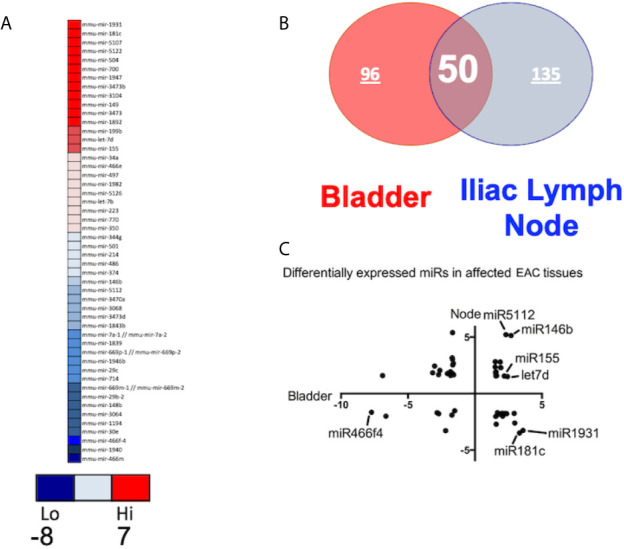
Differentially expressed miRs (DEMs) between the bladder and iliac lymph nodes in a mouse model of experimental IC. **(A)** Top DEMs diverge between tissue types and controls. **(B)**. Urinary bladder and iliac lymph nodes DEMs share similar distribution with 50 overlapping miRs. **(C)** Differential expression of miRs in urinary bladder *versus* iliac lymph nodes. Normalized in Log2 scale FC.

After global normalization of the raw data, from UB *vs* control and ILNs *vs* control, we identified several miRs that were differentially expressed in mice with IC as compared to controls (**Figures 1C**, **D**). During IC, several miRs that overlap with disease-associated networks between bladder and ILNs were significantly (>2-fold) up-or down-regulated (**Figure 2A**). From this profile, we identified a set of altered 50 miRs that are common to both UB and ILNs (**Figure 2B**). Based on ingenuity pathways analysis, we hypothesize that a specific set of altered miRs might be involved in regulating inflammatory responses during IC in mice (**Figure 2C**).

**Figure 3:**
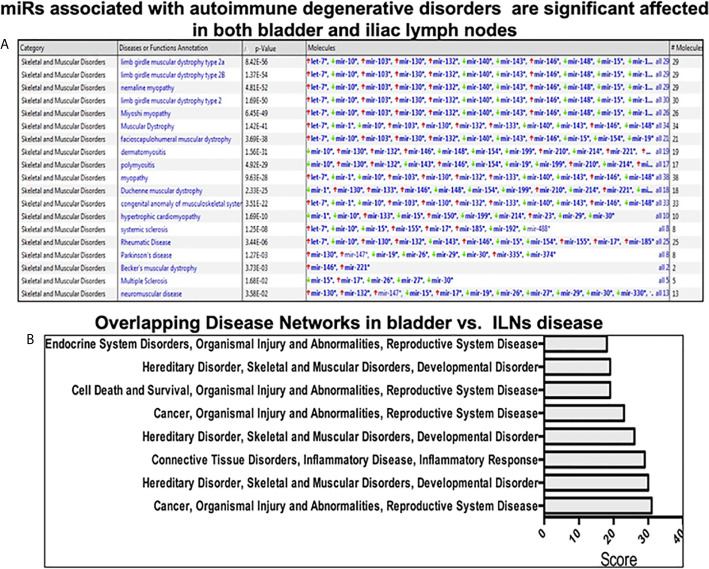
Pathway analysis using Ingenuity software during IC development. **(A)** The upper panel shows miRs associated with the autoimmune degenerative disorder in the urinary bladder and iliac lymph nodes. **(B)** Top ingenuity pathways analysis (IPA) overlapping disease-associated networks between tissue types in both urinary bladder and iliac lymph nodes.

In this study, we observed upregulation of miR-146, which negatively regulates microglial pro-inflammatory cytokines downstream of NF-κB, and miR-148, which is present in T cells from HIV-associated autoimmunity and Crohn’s disease (**Figure 3**). We also observed overexpression of miR-148, which predisposes the host to autoimmune diseases, including systemic lupus erythematosus (SLE) (17). We have also observed downregulation in the ILNs of miR-181, which exhibits anti-inflammatory effects during inflammation (18–20). Together, these results suggest that IC development and progression may be related to differential expression of a specific subset of miRs that modulate the disease severity and function *via* various inflammatory pathways.

### Validation of Differential miR Expression in UB and ILNs During IC

**Figure 4:**
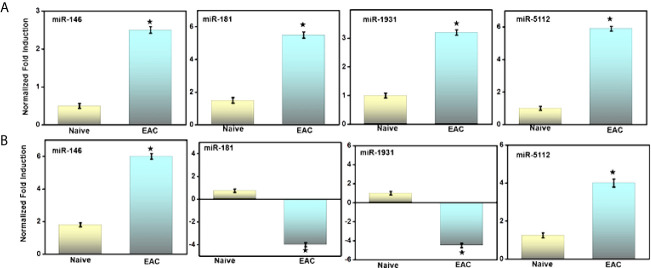
Validation of selected miRs by RT-PCR analysis after IC development. **(A)** As shown in the figure is quantitative RT-PCR verifying upregulation of miRs-146, -181, -1931, and -5112, in urinary bladder after IC development. **(B)** Similarly, the relative expression of iliac lymph nodes miRs 146, -5112 are upregulated, while miRs 181, -1931 are downregulated at iliac lymph nodes sites. Data are expressed as mean ± S.E.M. Statistical significance was calculated using the student’s t-test. *P > 0.05 control group alone *versus* IC group.

Next, to validate the alteration of miRs expression in the UB and ILNs during the progression of autoimmune IC. At the experimental endpoint, we isolated the immune cells from UB and ILNs and performed a quantitative RT-PCR analysis to confirm the altered expression of this miRs. In both UB and ILNs, miR-146 and miR-5112 increased by approximately 2.5- and 5-fold, respectively, compared to the levels detected in naïve control animals (**Figure 4**). We also observed a 5-fold increase in the expression of miR-181 in the UB of mice with IC, but miR-181 levels decreased 3-fold in ILNs (**Figure 4**). We observed a similar change in miR-1931 in both UB and ILNs, although any immunoregulatory role for miR-1931 has yet to be established. MiR-5112 was also upregulated in both UB and ILNs of IC mice as compared to control mice. Taken together, these data suggest that differential changes in UB and ILNs miRs during IC might be crucial for the disease progression.

### Changes in Pro-Inflammatory Cytokines and Chemokines During IC Progression

**Figure 5:**
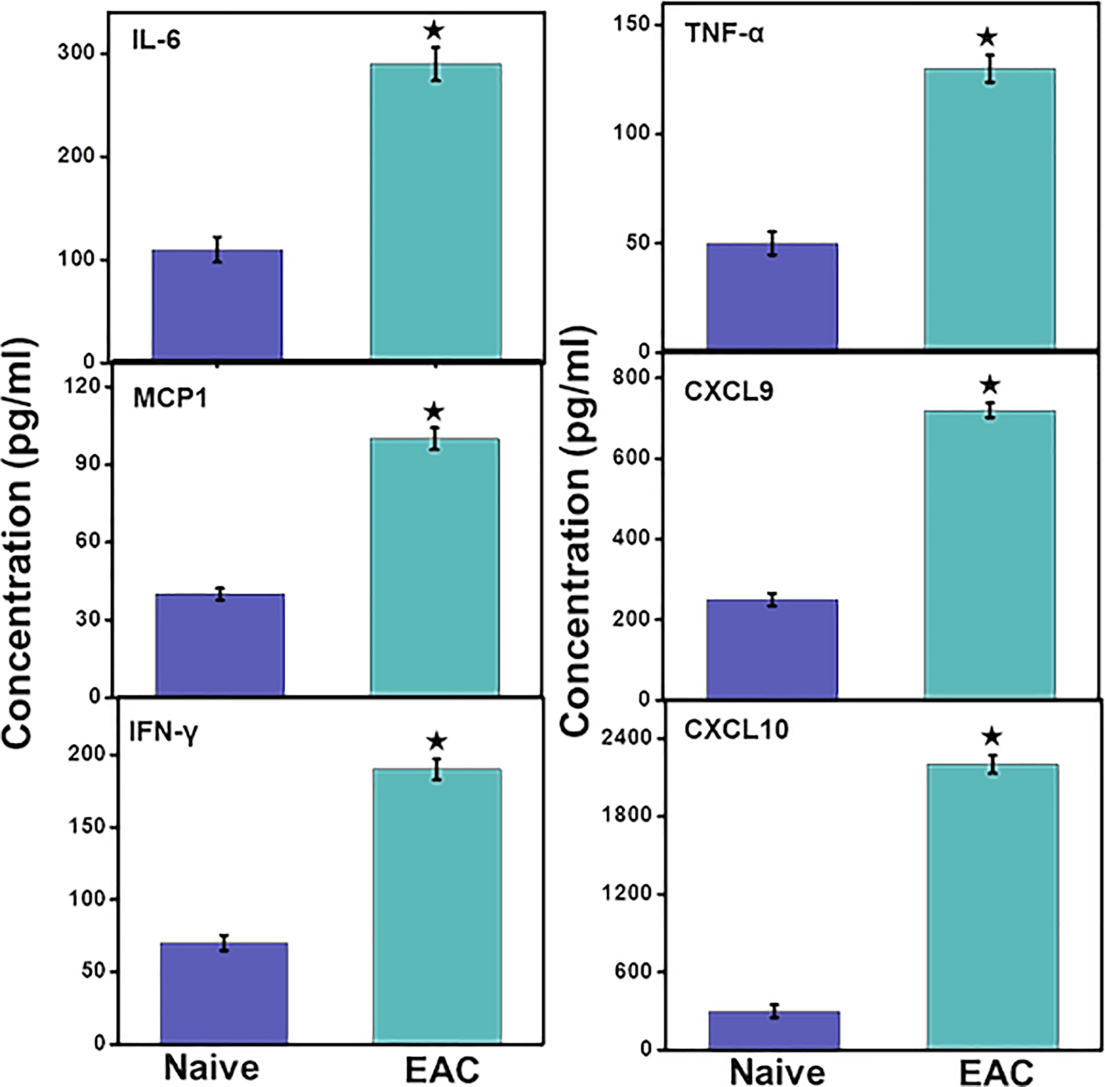
IC induction induces levels of systemic pro-inflammatory and inflammatory cytokines and chemokines. After mice were sacrificed, serum levels of IL-6, MCP-1, CXCL9, CXCL10, TNF-α and IFN-γ were determined by a Bio-Rad ELISA multiplex kit capable of detecting >10 pg/ml of these analytes. The data presented are the cumulative mean concentrations of these cytokines ± in three separate experiments. Asterisks (*) indicate statistically significant differences (p < 0.01) between IC and naïve control groups.

Levels of the systemic pro-inflammatory cytokines IL-6 and TNF-α levels increased significantly (p< 0.01) in IC mice as compared to control mice. These data suggest that the development of IC is associated with increased levels of systemic inflammation that may further promote immune cell infiltration at the site of inflammation in UB. Furthermore, we observed a significant (p<0.01) increase in different pro-inflammatory chemokines CXCL9, CXCL10, and monocyte chemoattractant protein 1 (MCP1) that regulate the immune cell chemotaxis and infiltration to the site of inflammation, in this case, the UB (**Figure 5**). This systemic increase in the pro-inflammatory cytokines and chemokines suggests that IC may induce systemic inflammation that may further aggravate the IC symptoms *via* recruiting pro-inflammatory immune cells. Also, the systemic increase in levels of interferon-γ (IFN-γ) in IC significantly (p<0.01) indicates the systemic activation of immune cells that are responsible for its production and induce inflammation.

### Changes in miRs Expression Correlates With the Severity of IC

**Figure 6:**
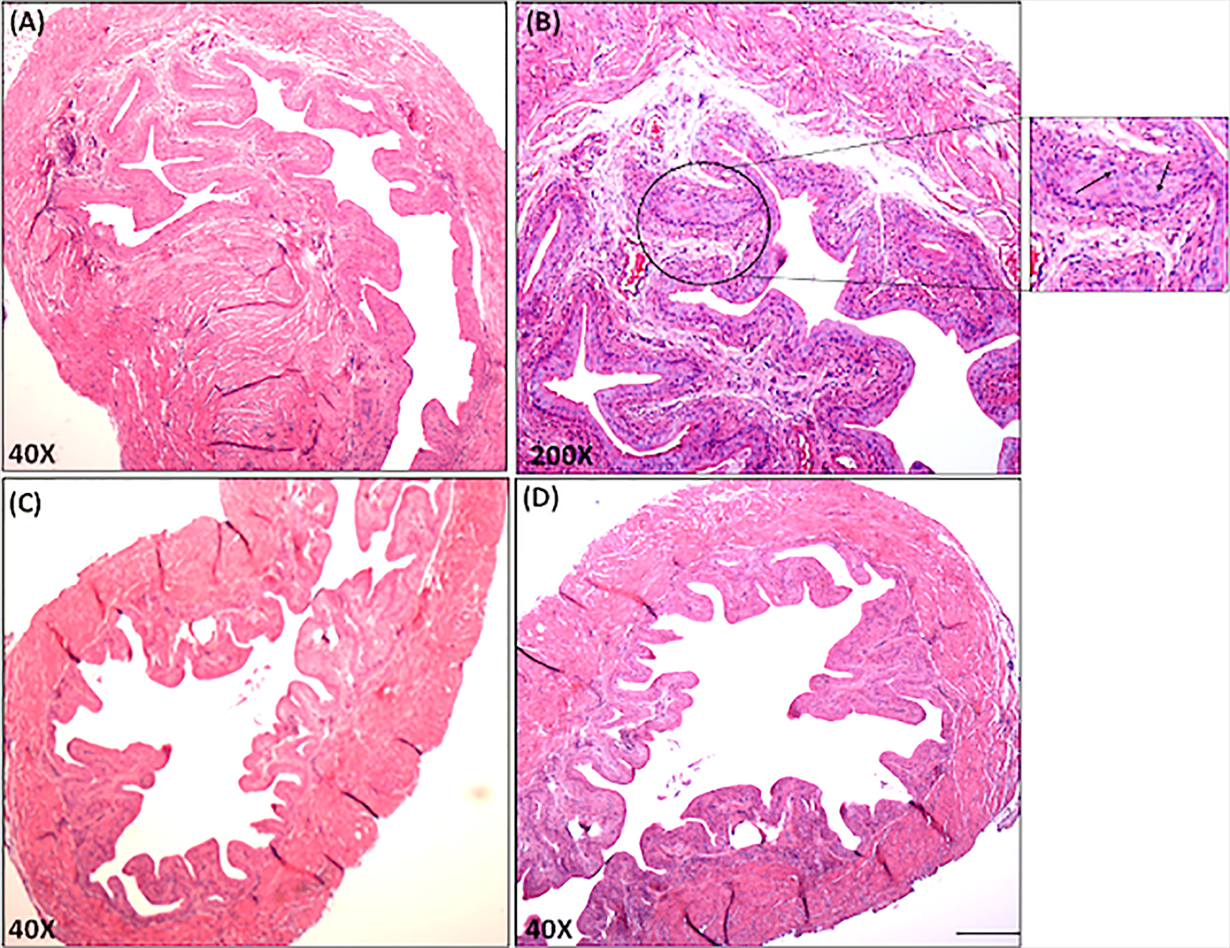
Histopathological changes in the urinary bladder of chronic IC development. Urinary bladder histopathological sections from control and IC are shown at different magnifications (**A, C, D** X40; **B**, X200). Upper Panels A and B shows sections from IC-induced mice illustrate inflamed bladders characterized by differences in mucosal wall thickness, narrow bladder space, enlargement of the mucosal layer and leukocyte infiltration (arrows in the inset). These histological features of IC model share similarities with clinical IC pathology. Panel C and D shows normal bladder space, no cellular infiltrates, and normal epithelial cells. Representative pictures are from 3 independent experiments using 6 mice in each group. Bar 200 μm **(A–D)**.

It remains to be determined whether IC symptoms are associated with the differential changes in miRs that we observed. In control mice, the stroma of the UB was attached, as manifested by a decrease in UB mass with few leukocytes’ infiltrates (**Figures 6C**, **D**). We also calculated a lower disease score in these naïve control mice than that observed for IC mice (**Table 1**). In contrast, the UB mucosa of IC mice showed pathological findings similar to those seen with clinical IC, characterized by loose connective tissue with extensive mixed leukocyte infiltrates primarily comprised of immune cells like T, mast, and neutrophils (**Figures 6A**, **B**). Taken together, these data suggest that differential expression of a subset of miRs may alter the severity of IC in mice.

**Table 1:** Histological evaluation of mice with or without IC.

Group	Number of Mice	IC Disease Score (0 – 4)
IC	10	2.41± 0.72*
Control mice	10	0

The UB disease score was determined by histopathological examination at the end of the experiment. UBs were fixed, sectioned at 6 µm, and stained with hematoxylin and eosin, examined microscopically at 10X and 40X magnification, and scored for the severity of IC. The studies were performed in triplicate and the data represent the mean and percent change ± SD. Differences between IC and naïve groups were considered significant when p < 0.01 (*).

## Discussion

IC is a chronic disease that affects 1-2% of the population that is seen more frequently in women than in men. Various chronic pain conditions, including irritable bowel syndrome (IBS), may also serve as causal factors for IC. The treatment of IC is hampered by the lack of knowledge about the exact etiology and pathogenesis of IC. Despite decades-long efforts by NIH and other organizations, only little impact has been made on the diagnosis, prevention, and/or effective treatment of IC. Therefore, in the present study, we investigated whether the dysregulation of UB and ILNs miRs play any role in the pathogenesis of IC, using the experimental autoimmune mouse model of IC that displays an autoimmune phenotype with histological alterations confined to the urinary bladder. In this study, we demonstrated that during IC progression, a set of miRs exhibited dysregulated expression which correlated with increased levels of inflammatory cytokines and chemokines and with histological changes in the UB, indicating a potential role for this miRs in the pathogenesis of IC.

miRs regulate multiple genes by binding to target mRNAs, thereby controlling the stability and translation of protein-coding mRNAs (21, 22). Further, miRs expressed in many developing peripheral tissues can regulate inflammation (23, 24). In this study, we observed upregulation of miR-146 in the UB and ILNs of IC mice as compared to control mice. It has been shown that miR-146 is highly expressed in the synovial fluid of RA patients and its upregulation is controlled through pro-inflammatory cytokines TNF-α and IL-1α (25). Towards this, we also noticed significantly increased systemic levels of TNF-α in IC mice, an observation that supports the previous finding that miRs-146 expression is the regulator of pro-inflammatory cytokines during chronic inflammatory or autoimmune diseases. Furthermore, TNF-α can induce miR-146 expression *via* IRAK1 inflammatory pathways (26–28). Hence, we propose that because the miR-146 expression is directly associated with levels of pro-inflammatory cytokines in inflammatory conditions, it might play a role in the induction of IC.

We observed that expression of miR-5112 increased in the UB and ILNs of IC mice as compared to control mice. miR-5112 is upregulated in breast cancer tissues, where it targets the cytoplasmic polyadenylation element-binding protein 1 (CPEB1) that serves as an internal inhibitor of the pro-inflammatory cytokine IL-6 (29, 30). Inhibition of IL-6 expression by CPEB1 mainly involves downregulation of transforming growth factor beta-activated kinase 1 (TAK1) production thereby impacting this inflammatory pathway and phenotype (31). Hence, an increase in the expression of miR-5112 will aggravate inflammation by inhibiting CPEB1 expression to increase IL-6 production. This corresponds well with our finding in the present study that IL-6 is upregulated in IC relative to the control.

Further, we also observed an increase in miR-181 in the UB and ILNs of IC mice compared to the control. MiR-181 plays a pro-inflammatory role in hypoxia/reoxygenation injury through non-receptor tyrosine phosphatase 4 (PTPN4) heart cells (32). MiR-181 also regulates T cell-based immune responses *via* its crucial role in the activation of CD4^+^ T cells (33). MiR-181 expression is well correlated with the expression of pro-inflammatory cytokines (IL-β, IL-6, and TNF-α), and its inhibition prevents induction of NF-κB-dependent pro-inflammatory cytokine generation *in vitro*. Levels of miR-181 are also increased in the cerebrospinal fluid (CSF) of multiple sclerosis (MS) patients, suggesting that it may play a pro-inflammatory and therefore has the potential to serve as a pro-inflammatory marker (34). Taken together, our findings suggest that upregulation of several pro-inflammatory miRs (miR-146a, miR-5112, and miR-181c) in the UB and ILNs in the mouse IC correlates with an increase in levels of IL-6 and TNF-α. Further mechanistic studies to either mimic or overexpress this IC-associated miRs will be required to decode the apparent miR-based regulation of pro-and anti-inflammatory signaling cascades to establish their roles in IC progression. However, these upregulated circulating miRs might also serve as biomarkers of the clinical progression of IC in patients.

Levels of the chemokines CXCL9, CXCL10, and MCP-1, which are involved in the chemoattraction of T cells and monocytes/macrophages at the site of inflammation (29, 35), also increased in experimental IC when compared to normal control mice. In this study, we observed an increase in the systemic interferon-γ (IFN-γ), CXCL9, and CXCL10 in IC, suggesting the activation of a Th1 pro-inflammatory immune response by interaction with CXCR3-expressing immune cells including T cells and macrophages. The increased levels of CXCL9 suggest the infiltration of macrophages in the inflamed UB of IC mice (36). Expression of MCP-1 promotes the recruitment of monocyte/macrophages, effector T cells, and dendritic cells (DC) at the site of inflammation (37, 38), which is consistent with the immune infiltration that we observed in the UB in mice with IC. Hence, infiltration of T cells and monocyte/macrophages in the UB of mice with IC leads to the release of pro-inflammatory mediators (cytokines, IFNs, and chemokines) to induce inflammatory damage and other IC-associated symptoms exhibited by IC patients. The experimental autoimmune IC model that we used in this study mimics many of the clinical (urinary frequency and decreased urine output per void) and histopathological features of human IC. This includes a decrease in bladder urothelial detachment, increased bladder permeability with epithelial leakage, lymphocyte infiltration, fibrosis, and edema (6, 39). However, it would be interesting the investigate the crosstalk between infiltrated immune cells and miRs overexpression during IC progression, because this model shares some of the features with clinical IC and shows promise for a future transitional outcome.

To the best of our knowledge, this report is the first of its kind to demonstrate the dysregulation in the expression of miRs in the UB and ILNs of IC in mice. We demonstrated that the expression of miR-146, miR-5112, and miR-181 increased in UB and ILNs of IC mice. Expression of these sets of miRs is well correlated with systemic pro-inflammatory markers (cytokines, IFNs, and chemokines) and a histopathological score of inflamed UB in mice with IC. It will be interesting to study further the interaction between infiltrated immune cells, expression of tissue miRs, and UBs epithelial cell crosstalk during IC progression. Further studies are also required to explore the use of these sets of miRs as biomarkers for clinical IC. Here we provided our initial findings to investigate the potential role of miRs in the development and progression of IC.

## Data Availability Statement

The datasets presented in this study can be found in online repositories. The names of the repository/repositories and accession number(s) can be found below: NCBI GEO; GSE177066.

## Ethics Statement

The animal study was reviewed and approved by The Institutional Animal Care and Use Committee (ICAUC) of the University of South Carolina School of Medicine approved this study from 2014 to 2017.

## Author Contributions

Author US conceived the ideas, wrote the manuscript, performed the work and data analysis. HS performed part of the work, VK and SK made the figures, performed data analysis, and assisted in the writing of the manuscript. All authors contributed to the article and approved the submitted version.

## Funding

This study was supported in part by grants from NIAID R01 AI140405 to US, at UTHSC in Memphis, TN.

## Conflict of Interest

The authors declare that the research was conducted in the absence of any commercial or financial relationships that could be construed as a potential conflict of interest.

## Abbreviations

IC, Interstitial cystitis; UB, Urinary bladder; ILNs, Iliac lymph nodes; PBS, Painful bladder syndrome; MicroRNAs, miRs; Ab, Antibody; EAC, Experimental Autoimmune cystitis; ELISA, Enzyme-linked immunosorbent assay; FBS, Fetal bovine serum; IFN, Interferon; NCBI, National Center for Biotechnology Information; PBS, Phosphate-buffered saline; RT, Room temperature; RT-PCR, Real time-polymerase chain reaction; SEM, Standard Error mean; TNF-α, Tumor necrosis factor α; MCP1, Monocyte Chemoattractant Protein 1; IL-6, Interleukin 6.

## References

[1] Nordling J Fall M Hanno P. Global Concepts of Bladder Pain Syndrome (Interstitial Cystitis). World J Urol (2012) 30(4):457–64. 10.1007/s00345-011-0785-x 22057291

[2] Droupy S. The Therapeutic Approach to Different Forms of Cystitis: Impact on Public Health. Urologia (2017) 84(Suppl 1):8–15. 10.5301/uj.5000262 28862726

[3] Jaini R Hannaman D Johnson JM Bernard RM Altuntas CZ Delasalas MM. Gene-Based Intramuscular Interferon-Beta Therapy for Experimental Autoimmune Encephalomyelitis. Mol Ther (2006) 14(3):416–22. 10.1016/j.ymthe.2006.04.009 16782409

[4] Jane-wit D Yu M Edling AE Kataoka S Johnson JM Stull LB. A Novel Class II-Binding Motif Selects Peptides That Mediate Organ-Specific Autoimmune Disease in SWXJ, SJL/J, and SWR/J Mice. J Immunol (2002) 169(11):6507–14. 10.4049/jimmunol.169.11.6507 12444161

[5] Altuntas CZ Johnson JM Tuohy VK. Autoimmune Targeted Disruption of the Pituitary-Ovarian Axis Causes Premature Ovarian Failure. J Immunol (2006) 177(3):1988–96. 10.4049/jimmunol.177.3.1988 16849513

[6] Lin YH Liu G Kavran M Altuntas CZ Gasbarro G Tuohy VK. Lower Urinary Tract Phenotype of Experimental Autoimmune Cystitis in Mouse: A Potential Animal Model for Interstitial Cystitis. BJU Int (2008) 102(11):1724–30. 10.1111/j.1464-410X.2008.07891.x 18710451

[7] Singh UP Singh NP Guan H Hegde VL Price RL Taub DD. The Severity of Experimental Autoimmune Cystitis can be Ameliorated by Anti-CXCL10 Ab Treatment. PloS One (2013) 8(11):e79751. 10.1371/journal.pone.0079751 24278169 PMC3836899

[8] Gamper M Viereck V Eberhard J Binder J Moll C Welter J. Local Immune Response in Bladder Pain Syndrome/Interstitial Cystitis ESSIC Type 3C. Int Urogynecology J (2013) 24(12):2049–57. 10.1007/s00192-013-2112-0 PMC3838592 23670165

[9] Malik ST Birch BR Voegeli D Fader M Foria V Cooper AJ. Distribution of Mast Cell Subtypes in Interstitial Cystitis: Implications for Novel Diagnostic and Therapeutic Strategies? J Clin Pathol (2018) 71(9):840–4. 10.1136/jclinpath-2017-204881 29764932

[10] Lin TJ Garduno R Boudreau RT Issekutz AC. Pseudomonas Aeruginosa Activates Human Mast Cells to Induce Neutrophil Transendothelial Migration *via* Mast Cell-Derived IL-1 Alpha and Beta. J Immunol (2002) 169(8):4522–30. 10.4049/jimmunol.169.8.4522 12370389

[11] Vincent-Schneider H Thery C Mazzeo D Tenza D Raposo G Bonnerot C. Secretory Granules of Mast Cells Accumulate Mature and Immature MHC Class II Molecules. J Cell Sci (2001) 114(Pt 2):323–34. 10.1242/jcs.114.2.323 11148134

[12] Wu F Zikusoka M Trindade A Dassopoulos T Harris ML Bayless TM. MicroRNAs Are Differentially Expressed in Ulcerative Colitis and Alter Expression of Macrophage Inflammatory Peptide-2 Alpha. Gastroenterology (2008) 135(5):1624–1635 e1624. 10.1053/j.gastro.2008.07.068 18835392

[13] Muljo SA Ansel KM Kanellopoulou C Livingston DM Rao A Rajewsky K. Aberrant T Cell Differentiation in the Absence of Dicer. J Exp Med (2005) 202(2):261–9. 10.1084/jem.20050678 PMC2212998 16009718

[14] Singh UP Murphy AE Enos RT Shamran HA Singh NP Guan H. miR-155 Deficiency Protects Mice from Experimental Colitis by Reducing T Helper Type 1/Type 17 Responses. Immunology (2014) 143:478–89. 10.1111/imm.12328 PMC4212960 24891206

[15] Sakthivel SK Singh UP Singh S Taub DD Novakovic KR Lillard JW Jr. CXCL10 Blockade Protects Mice From Cyclophosphamide-Induced Cystitis. J Immune Based Ther Vaccines (2008) 6(1):6. 10.1186/1476-8518-6-6 18957084 PMC2583981

[16] Shamran H Singh NP Zumbrun EE Murphy A Taub DD Mishra MK. Fatty Acid Amide Hydrolase (FAAH) Blockade Ameliorates Experimental Colitis by Altering microRNA Expression and Suppressing Inflammation. Brain Behav Immun (2017) 59:10–20. 10.1016/j.bbi.2016.06.008 27327245 PMC5154806

[17] Gonzalez-Martin A Adams BD Lai M Shepherd J Salvador-Bernaldez M Salvador JM. The microRNA miR-148a Functions as a Critical Regulator of B Cell Tolerance and Autoimmunity. Nat Immunol (2016) 17(4):433–40. 10.1038/ni.3385 PMC4803625 26901150

[18] Ralfkiaer U Hagedorn PH Bangsgaard N Lovendorf MB Ahler CB Svensson L. Diagnostic microRNA Profiling in Cutaneous T-Cell Lymphoma (CTCL). Blood (2011) 118(22):5891–900. 10.1182/blood-2011-06-358382 PMC3342856 21865341

[19] Su R Lin HS Zhang XH Yin XL Ning HM Liu B. MiR-181 Family: Regulators of Myeloid Differentiation and Acute Myeloid Leukemia as Well as Potential Therapeutic Targets. Oncogene (2015) 34(25):3226–39. 10.1038/onc.2014.274 25174404

[20] Huang X Schwind S Santhanam R Eisfeld AK Chiang CL Lankenau M. Targeting the RAS/MAPK Pathway With miR-181a in Acute Myeloid Leukemia. Oncotarget (2016) 7(37):59273–86. 10.18632/oncotarget.11150 PMC5312311 27517749

[21] Guo H Ingolia NT Weissman JS Bartel DP. Mammalian microRNAs Predominantly Act to Decrease Target mRNA Levels. Nature (2010) 466(7308):835–40. 10.1038/nature09267 PMC2990499 20703300

[22] Esteller M. Non-Coding RNAs in Human Disease. Nat Rev Genet (2011) 12(12):861–74. 10.1038/nrg3074 22094949

[23] Ambros V. The Functions of Animal microRNAs. Nature (2004) 431(7006):350–5. 10.1038/nature02871 15372042

[24] O’Connell RM Rao DS Baltimore D. microRNA Regulation of Inflammatory Responses. Annu Rev Immunol (2012) 30:295–312. 10.1146/annurev-immunol-020711-075013 22224773

[25] Nakasa T Miyaki S Okubo A Hashimoto M Nishida K Ochi M. Expression of microRNA-146 in Rheumatoid Arthritis Synovial Tissue. Arthritis Rheum (2008) 58(5):1284–92. 10.1002/art.23429 PMC2749927 18438844

[26] Taganov KD Boldin MP Chang KJ Baltimore D. NF-kappaB-Dependent Induction of microRNA miR-146, an Inhibitor Targeted to Signaling Proteins of Innate Immune Responses. Proc Natl Acad Sci USA (2006) 103(33):12481–6. 10.1073/pnas.0605298103 PMC1567904 16885212

[27] Abou-Zeid A Saad M Soliman E. MicroRNA 146a Expression in Rheumatoid Arthritis: Association With Tumor Necrosis Factor-Alpha and Disease Activity. Genet Test Mol Biomarkers (2011) 15(11):807–12. 10.1089/gtmb.2011.0026 21810022

[28] Xia P Fang X Zhang ZH Huang Q Yan KX Kang KF. Dysregulation of Mirna146a Versus IRAK1 Induces IL-17 Persistence in the Psoriatic Skin Lesions. Immunol Lett (2012) 148(2):151–62. 10.1016/j.imlet.2012.09.004 23018031

[29] Lee JH Kim B Jin WJ Kim HH Ha H Lee ZH. Pathogenic Roles of CXCL10 Signaling Through CXCR3 and TLR4 in Macrophages and T Cells: Relevance for Arthritis. Arthritis Res Ther (2017) 19(1):163. 10.1186/s13075-017-1353-6 28724396 PMC5518115

[30] Groppo R Richter JD. CPEB Control of NF-kappaB Nuclear Localization and Interleukin-6 Production Mediates Cellular Senescence. Mol Cell Biol (2011) 31(13):2707–14. 10.1128/MCB.05133-11 PMC3133380 21536657

[31] Ivshina M Alexandrov IM Vertii A Doxsey S Richter JD. CPEB Regulation of TAK1 Synthesis Mediates Cytokine Production and the Inflammatory Immune Response. Mol Cell Biol (2015) 35(3):610–8. 10.1128/MCB.00800-14 PMC4285422 25452303

[32] Wang S Ge L Zhang D Wang L Liu H Ye X. MiR-181c-5p Promotes Inflammatory Response During Hypoxia/Reoxygenation Injury by Downregulating Protein Tyrosine Phosphatase Nonreceptor Type 4 in H9C2 Cardiomyocytes. Oxid Med Cell Longev (2020) 2020:7913418. 10.1155/2020/7913418 32774684 PMC7399766

[33] Xue Q Guo ZY Li W Wen WH Meng YL Jia LT. Human Activated CD4(+) T Lymphocytes Increase IL-2 Expression by Downregulating microRNA-181c. Mol Immunol (2011) 48(4):592–9. 10.1016/j.molimm.2010.10.021 21112091

[34] Kramer S Haghikia A Bang C Scherf K Pfanne A Duscha A. Elevated Levels of miR-181c and miR-633 in the CSF of Patients With MS: A Validation Study. Neurol Neuroimmunol Neuroinflamm (2019) 6(6):e623. 10.1212/NXI.0000000000000623 31575652 PMC6812730

[35] Loos T Dekeyzer L Struyf S Schutyser E Gijsbers K Gouwy M. TLR Ligands and Cytokines Induce CXCR3 Ligands in Endothelial Cells: Enhanced CXCL9 in Autoimmune Arthritis. Lab Investig J Tech Methods Pathol (2006) 86(9):902–16. 10.1038/labinvest.3700453 16847431

[36] House IG Savas P Lai J Chen AXY Oliver AJ Teo ZL. Macrophage-Derived CXCL9 and CXCL10 Are Required for Antitumor Immune Responses Following Immune Checkpoint Blockade. Clin Cancer Res (2020) 26(2):487–504. 10.1158/1078-0432.CCR-19-1868 31636098

[37] Carr MW Roth SJ Luther E Rose SS Springer TA. Monocyte Chemoattractant Protein 1 Acts as a T-Lymphocyte Chemoattractant. Proc Natl Acad Sci USA (1994) 91(9):3652–6. 10.1073/pnas.91.9.3652 PMC43639 8170963

[38] Xu LL Warren MK Rose WL Gong W Wang JM. Human Recombinant Monocyte Chemotactic Protein and Other C-C Chemokines Bind and Induce Directional Migration of Dendritic Cells *In Vitro* . J Leukoc Biol (1996) 60(3):365–71. 10.1002/jlb.60.3.365 8830793

[39] Altuntas CZ Daneshgari F Sakalar C Goksoy E Gulen MF Kavran M. Autoimmunity to Uroplakin II Causes Cystitis in Mice: A Novel Model of Interstitial Cystitis. Eur Urol (2011) 61(1):193–200. 10.1016/j.eururo.2011.06.028 21719190 PMC3226908

